# Supplementary material for: Prevalence and determinants of anemia among young (15–24 years) women in Ethiopia: A multilevel analysis of the 2016 Ethiopian demographic and health survey data
Source: PLoS One. 2020 Oct 30;15(10):e0241342. doi: 10.1371/journal.pone.0241342 (PMC7599045; doi:10.1371/journal.pone.0241342)
Supplement: Attachment — Submitted filename: Comments on the paper on anemia in young persons in Ethiopia.docx [file pone.0241342.s001.docx]

Prevalence and determinants of Anemia among young (15-24 years) women in Ethiopia; A multilevel analysis of the 2016 Ethiopian Demographic and Health Survey data

The authors present findings from an analysis of data to determine the prevalence of anemia young women who participated in the 2016 Demographic Health Survey in Ethiopia. The key findings are the prevalence and identification of determinants of anemia among the study participants. The main strength of the study is the large sample size of women whose data were analyzed. However, the paper has several issues that the authors need to address before the paper can be suitable for publication in the journal.

The first issue is that the paper would need substantial English language editing; there are many editorial errors in the paper. The specific issues that require revision in the different components of the paper are provided below:

**Abstract**

1. The statement that there is a high prevalence of anemia in young persons (line 29) as a justification for the study is contradictory because If there is already high prevalence of anemia among young women why have the authors conducted another study among this same population? This should be clarified.
2. The statement ‘The overall prevalence of anemia among young women were 21.7% (line 40) should read ‘…was 21.7%’.

**Background**

1. There is need to clarify that the vulnerable population being referred to in line 69 are female adolescents and young women; this is necessary because male adolescents also suffer from anemia. This point should be clarified throughout the manuscript.
2. The statement ‘over half of young women worldwide are suffered...’ line 75 should read ‘… have suffered’
3. The statement ‘…and African as well...’ line 76 is not clear and should be revised.
4. If the statement ‘approximately one quarter of adolescents…’ line 78 is referring to female adolescents this should be clarified
5. There are many repetitive statements about the fact that sub-Sahara Africa has contributed to the high burden of anemia, this should be revised.
6. The authors have listed the socio-demographic factors (lines 89-92) that contribute to anemia in Ethiopia, but examples are not provided to illustrate this point. Some examples should be provided to illustrate this point.
7. The statement ‘Due to rapid growth…’ line 93 is a repetition because this point has been made earlier
8. The sentence ‘…and up to our knowledge’ line 96 should read ‘…to our knowledge’
9. The data for the study were extracted from the 2016 EDHS; is this the latest survey in the country? Have there been other surveys since the one in 2016? If there has been a more recent survey, the authors need to justify why they have used the 2016 survey as source of data for this study. This is should be clarified

**Methods**

1. An important information missing about the study area is the population of Ethiopia and more importantly the population of young persons in the country.
2. The reference to altitude and smoking (line 132) is not clear; how do these variables relate to the issue investigated. This should be clarified.

**Results**

1. The statement ‘of women were not had work’ line 170-171 is not clear and should be revised
2. The word ‘afar’ line 183 should read ‘Afar’
3. The use of the phrase ‘followers of Muslim and protestant religion’ line 203 are not clear. A better phrase may be people who practice Islam or Moslems and protestant Christians. This should be revised.
4. The statement ‘… with their counterparts’ line 207 should read ‘with their counterparts who do not’
5. The figure showing prevalence of anemia should have a number and reference should be made to it in the text

**Discussion**

1. I suggest that the authors provide the figure being referred to in the statement ‘but greater than the previous reported in our country’ line 218
2. The authors should give examples of the food types being referred to in line 228 and support this statement with appropriate reference
3. The statement i.e. barefooted line 241 should be revised to read walking barefooted
