# Supplementary material for: Prevalence and determinants of anemia among young (15–24 years) women in Ethiopia: A multilevel analysis of the 2016 Ethiopian demographic and health survey data
Source: PLoS One. 2020 Oct 30;15(10):e0241342. doi: 10.1371/journal.pone.0241342 (PMC7599045; doi:10.1371/journal.pone.0241342)
Supplement: Attachment — Submitted filename: New comments on revised paper on anemia among young women in Ethiopia, 210920.docx [file pone.0241342.s003.docx]

Prevalence and determinants of Anemia among young (15-24 years) women in Ethiopia; A multilevel analysis of the 2016 Ethiopian Demographic and Health Survey data

There is substantial improvement in the presentation of the revised manuscript. The authors have addressed all the issues and queries I raised in my previous review of the manuscript. However, in re-reading the manuscript, I detected some minor editorial errors which require the authors attention.

1. In line 80, the word ‘where’ should be replaced by the word ‘with’ to make the sentence clearer.
2. The statement on line 133 and 134 should be presented in past tense ‘for pregnant women…was considered as anemic…’
3. In line 165, the first letter of the university should start with a capital letter, to read ‘University of Gondar’; the authors should also clarify if this institution is in Ethiopia.
4. The statement on 226 needs revision; it should read ‘…greater than the 15.6% anemia found among young women in Rwanda’
5. The author should clarify which country is being referred to on line 227; is this Ethiopia or Rwanda? This should be revised.
6. The statement on line 262 should read ‘…which are common in lowland areas’
7. The word on line 330 should read ‘References’ not ‘reference’
