# Supplementary material for: Prevalence and determinants of anemia among young (15–24 years) women in Ethiopia: A multilevel analysis of the 2016 Ethiopian demographic and health survey data
Source: PLoS One. 2020 Oct 30;15(10):e0241342. doi: 10.1371/journal.pone.0241342 (PMC7599045; doi:10.1371/journal.pone.0241342)
Supplement: Attachment — Submitted filename: Response to reviewer.docx [file pone.0241342.s004.docx]

Date: October/2020

**Author's point to point response reviewers comments**

**Title:** Prevalence and determinants of Anemia among young (15-24 years) women in Ethiopia; A multilevel analysis of the 2016 Ethiopian Demographic and Health Survey data

**Manuscript number:** PONE-D-20-18515

**Subject: Submitting a revised version of the manuscript**

We would like to thank the reviewers and editor for sharing their view and novel scholarly experiences. Still, the comments are very imperative which we strongly believe in improving the manuscript. We try to address all the comment raised by the revivers line by line in the main document. The point-by-point responses for each of the comments, questions, and the revised manuscript are provided in the attached documents.

Thank you for considering our manuscript again.

**Response to reviewers comment**

**Response to reviewer#2**

**Reviewer 2 comment 1**

1. Thanks for the major revisions to the manuscript. However I still have major concerns see them in detailed explained hereafter. But most important; I still mis out in your introduction and discussion. Why your study was essential to do? And what does it bring clearly new to this research field? Your methods and numbers are good but without that message very clear I am doubting for the eligibility for international readers. Please adjust that as you stated nicely in the explanations given to my comments.

**Author’s response:** Dear reviewer thank you for your important concern. We have included the information we have provided in response to the introduction part of the revised version of the manuscript. We adjust the introduction part by incorporating the information about importance of conduction this study among young women. In addition the reason for conducting this study was as anemia in young women is a serious condition which impedes them from reaching their full potential by reducing educational achievement, labor productivity as well as their cognitive capacity and affect their mental health (see page 4 line 88-90).

2. Reviewer 2 Comment 1 eligibility for international readers; Thanks for your clear response please add this information to the introduction

**Author’s response:** dear reviewer thank you for your comment: we have taken the comment and included it in the introduction part of the revised manuscript. We have incorporated an important information which justify the reason for this study among those populations.

3. Suggestion 1: change in the abstract line 27 particular in including

**Author’s response: thank you for your comment:** we taken the comment and replaced the word particular with including in the revised version of the manuscript.

4. Suggestion 2: line 99 add the age range of productive age (define)

**Author’s response: thank you for your comment:** we take the comment and we have add the age range in the revised version of the manuscript. As we have indicted in the revised version of the manuscript reproductive age women includes women of from 15 to 49 years of age, while young women include women of 15 to 24 years of age.

5. Suggestion 3: so explain why these two groups are different. Reproductive age and young woman and why your study is needed.

**Author’s response: thank you for your comment:** as we have included in the revised version of the manuscript the age range for reproductive age women ranges from 15 to 49 years of age and those for young women are from 15 to 24 years of age as described by the United nation. Usually as women become older their extreme demand for those micronutrients may decreased and they have less risk of developing those micronutrients deficiency associated health problems including anemia. When considering young aged women (15 to 24 years) these population are at an alarming rate of their body growth and they have an excesses biological demand for micronutrients and other nutrients that neccesiciate for their body growth. For the above mentioned reason we have conducted this study to asses anemia prevalence and associated factor among young women. This information is stated throughout the background section of the revised manuscript.

**6.** Suggestion 4: line 100” determinates in Ethiopia” change to in Africa (or LMIC) including	Ethiopia

**Author’s response:** thank you for your comment**:** we have corrected it per your recommendation in the revised version of the manuscript.

**Reviewer 2 comment 9**

**1.** Line 73 young woman and adolescents’ female. Change in including adolescent females

**Author’s response**: dear reviewer thank you for your comment: we accepted your comment and corrected it in the revised version of our manuscript.

**Reviewer 2 Comment 15:**

1. See like the statement at comment 1. Clear clarification but please add this information to your introduction. To me it is still not clear why this study needed to be done. Please clarify clearly in the introduction and aim of the study.

**Author’s response**: dear reviewer thank you for your comment: we have put these clarification in the introduction part of the revised version of the manuscript. Also as we have stated in the revised version of the manuscript young women (15-24) are highly vulnerability to anemia compared with older women because of their extra demand for micro and macronutrients associated with their physiological body growth (see line 99-106, page 5).

**Reviewer 2 Comment 22:**

1. Please state this calculation in the method section. Because you still used it and now it is taken out fully. That is incorrect. Please change.

**Author’s response**: thank you for raising this important issue: we have taken the comment and we have included the hemoglobin adjustment calculation in the revised version of the manuscript.

Reviewer 2 Comment 31:

1. Line 181-184: please add the CI to the groups you compare with as well and the p value. Now the sentence is not complete. Or take out all the CI and just give the P value. But the statement reads incorrect in the way it is stated now.

**Author’s response**: thank you for raising this important issue. Here we have put the descriptive percentage of the overall prevalence of anemia with 95%CI and the range in anemia prevalence among different region of Ethiopia. We didn’t have made any comparison here rather we indicate the variability in the range of prevalence of anemia among different region of Ethiopia. About the comparison of anemia in different region of Ethiopia we have put it in the multilevel analysis part (see line 226-229, page 10, see table 3, page 24-26). Here as we put in table 3 significant level of anemia among different region of Ethiopia were compared with Addis Ababa and we put the p value and 95%CI for AOR to indicate region that have statistical significant higher anemia level.

2. Please add the CI to figure 3

**Author’s response:** dear reviewer thank you for this important comment: we have included the 95%CI for the prevalence of anemia for each regions to figure 3 in the revised version of the manuscript (see figure 3).

**Reviewer 2 Comment 32:**

1. Please add in section 181-184 to which area it was compared. Compared to addis… etc.

**Author’s response;** dear reviewer thank you for your comment: here we have put the overall prevalence of anemia with 95%CI in Ethiopia and the prevalence in the nine region and two administrative city. So we have simply put the range in the prevalence of anemia among young women in the different region of Ethiopia rather than making comparison. We have described it as the highest prevalence of anemia was in Somalia region among the nine region and two city administration and the lowest prevalence was among women of Addis Ababa, which shows the regional variation of anemia.

**Reviewer 2 Comment 34:**

1. This comparison to Timor is still out of place in my perspective. Please refrase. Either put this statement together with the comparison at line 225 or take out.

**Author’s response:** dear reviewer thank you for you important comment; here we have compared our finding with the finding of Armenia and Timor since this study had a similar finding with our study. Most other study we have compared with our finding was either greater than or smaller than the prevalence of anemia among young women reported in our study. We compared to this region since this region are one part of countries with low and middle socioeconomic status which had almost similar to African country.

**Reviewer 2 Comment 35:**

1. Thanks for this change. However to me it is still not clear why your study was important when you have these numbers already. Please rephrase and explain what your study showed more. Because in the way it reads now like you redid the study.

**Author’s response:** Thank you reviewer: the number we have put was the report from previous study in Ethiopia which was conducted using previous DHS data(EDHS 2011) and it didn’t assessed different factors that will influence anemia prevalence among young women. We have conducted this study using the most recent EDHS data (EDHS 2016) and we have also assessed different determinant that will potentially affect anemia prevalence among these population, which didn’t addressed in the previous study. In addition to that, the DHS data has a hierarchical structure, which violates the independent assumptions of the standard logistic regression model and a multilevel logistic regression analysis was implemented in this study where the previous study didn’t considered this.

**Reviewer 2 Comment 40:**

1. Line 275: add these variables which you stated in your explanation there aswell.

**Author’s response: dear reviewer thank you for raising this important issue:** we have included the variables we have stated in the explanation as the limitation of this study in the strength and limitation part of the revised version of the manuscript (see page 13 line 294-297).

**Response to reviewer#5**

1. In line 80, the word ‘where’ should be replaced by the word ‘with’ to make the sentence clearer.

**Author’s response:** dear reviewer thank you for your important comment**:** we have taken the comment and revised it in the revised version of the manuscript.

2. The statement on line 133 and 134 should be presented in past tense ‘for pregnant women…was considered as anemic…’

**Author’s response:** dear reviewer thank you for raising this important issue: we have presented it in the past tense form as indicated by your comment.

3. In line 165, the first letter of the university should start with a capital letter, to read ‘University of Gondar’; the authors should also clarify if this institution is in Ethiopia.

**Author’s response:** dear reviewer thank you for your comment: we have corrected the grammatical error in the revised version of the manuscript and we have also indicate that University of Gondar is found in Ethiopia.

4. The statement on 226 needs revision; it should read ‘…greater than the 15.6% anemia found among young women in Rwanda’

**Author’s response:** dear reviewer thank you for your comment: we have revised this statement in the revised version of the manuscript.

5. The author should clarify which country is being referred to on line 227; is this Ethiopia or Rwanda? This should be revised.

**Author’s response:** thank you for your very important comment: here we have to mean this variation of anemia prevalence might be explained by the variation in the socioeconomic and cultural difference among different countries of Africa and we have revised it in the revised version of our manuscript.

6. The statement on line 262 should read ‘…which are common in lowland areas’

**Author’s response:** thank you for your very important comment: we have taken the comment and corrected it in the revised version of the manuscript.

7. The word on line 330 should read ‘References’ not ‘reference’

**Author’s response:** thank you for your very important comment: we have taken the comment and we corrected the grammatical error in the revised version of the manuscript.
